# Ab externo canaloplasty results and efficacy: a retrospective cohort study with a 12-month follow-up

**DOI:** 10.1186/s40662-019-0134-5

**Published:** 2019-03-12

**Authors:** Iraklis Vastardis, Sofia Fili, Zisis Gatzioufas, Markus Kohlhaas

**Affiliations:** 1 St. Johannes Hospital, Eye clinic, Dortmund, Germany; 2 Basel University Hospital, Eye Clinic, Basel, Switzerland

**Keywords:** Canaloplasty, Schlemm’s canal surgery, POAG

## Abstract

**Background:**

The aim of this study is to review the outcomes of canaloplasty versus canaloplasty combined with phacoemulsification in a retrospective cohort study and to evaluate the efficacy of these methods in terms of intraocular pressure (IOP) lowering effect, postoperative complications and additional glaucoma surgery or reintroduction of medical therapy over a 12-month follow-up.

**Methods:**

In a retrospective cohort study, 602 eyes with primary open angle glaucoma (POAG) were treated with canaloplasty or canaloplasty combined with phacoemulsification. The results were evaluated separately in two main groups; group A canaloplasty (262 eyes) and group B canaloplasty combined with phaco (322 eyes). Each group was then subdivided into three additional groups according to the severity of glaucoma. The criteria for successful treatment were evaluated between three IOP ranges; IOP ≤ 16 mmHg, 18 mmHg and 21 mmHg. Complete success was considered the percentage of eyes reaching target IOP with no medication and partial success with medication. Groups A and B subgroups were compared using the Kaplan Meier test. Mean IOP, reduction of antiglaucoma agents and additional IOP lowering methods were also evaluated. The follow-up time was 12 months. Statistical significance was set at *p* < 0.05.

**Results:**

An incomplete intraoperative cannulation of Schlemm’s canal resulting in conversion to other glaucoma surgery occurred in 18 eyes (2.99%). In both of the main groups, postoperative hyphema, descemet membrane detachment and transient IOP rise were the most common postoperative complications. The mean IOP in group A and subgroups at 12 months was 13.26 ± 4.5 mmHg, 15.19 ± 3.97 mmHg and 18.09 ± 3.75 mmHg. Respectively in group B mean IOP was 14.51 ± 4.69 mmHg, 14.40 ± 4.11 mmHg and 14.25 ± 2.76 mmHg. Complete success was achieved in group A in 69.19, 74.51 and 74.31% of eyes. In group B complete success was achieved in 81.60, 77.33 and 83.33% of eyes respectively. Kaplan Meier between groups A and B was statistically significant for IOP ≤ 16 mmHg and IOP ≤ 21 mmHg (*p* = 0.0041 and *p* = 0.0312), but not for IOP ≤ 18 mmHg (*p* = 0.6935). Partial success for IOP ≤ 16 mmHg was 95.23 and 92.26%, for IOP ≤ 18 mmHg was 91.66 and 90.47% and for IOP ≤ 21 mmHg, 90.00 and 93.10%, in groups A and B respectively. Twenty-three eyes received additional surgery (3.93%), 10 trabeculectomies and 2 cyclophotocoagulation in group A, and 9 trabeculectomies and 2 cyclophotocoagulation in group B.

**Conclusion:**

Canaloplasty and canaloplasty combined with phacoemulsification significantly lower the IOP and have a lower postoperative complication rate. Additional glaucoma surgery or medication following both procedures is necessary if target IOP is unsatisfactory. In this study, canaloplasty combined with phacoemulsification demonstrated superior success rate compared to canaloplasty alone.

## Background

Glaucoma is regarded as one of the major causes leading to blindness in both the developed and developing countries. Recent studies estimate that about 66 million people worldwide are affected with glaucoma, concluding that half of these cases remain undiagnosed [1]. In all cases of people affected with this condition, early diagnosis and sufficient reduction of the intraocular pressure (IOP), either with drug therapy or with surgery, has been proven to reduce rapid progression of visual field defects [2–4]. Drug therapy is administered as the treatment of choice upon diagnosis and early detection of primary open angle glaucoma (POAG) is crucial. Surgery is required when IOP is not manageable with topical therapy or when the socio-economic situation favors it as a tradeoff over years of expensive drug therapies. Trabeculectomy though, is still regarded as the golden standard. It is a filtering operation with excellent results in IOP reduction and is highly cost effective. As a surgical procedure, it relieves IOP by removing part of the eye’s trabecular meshwork and adjacent structures to allow drainage of aqueous humor from within the eye to underneath the conjunctiva where it is absorbed. However, its use has been highly correlated with severe, adverse sight threatening complications and difficult post-operative management [5, 6]. Non penetrating techniques were developed as controlled glaucoma incisional surgery without a full thickness opening. Canaloplasty in particular has been in the spotlight in the recent years as a viable option and considered the evolution of viscocanalostomy [7, 8]. It is a procedure designed to enhance and restore the eye’s natural drainage system and provide sustained reduction of intraocular pressure. After gaining access to Schlemm’s canal, a microcatheter is inserted inside and navigated 360° around it, dilating the main drainage channel and its smaller collector channels through the injection of viscoelastic. The catheter is then removed and a suture is placed within the canal and tightened. Aqueous outflow is restored by tensioning Schlemm’s canal with a 10.0 polypropylene suture. In this retrospective cohort study, 602 eyes with POAG were treated with canaloplasty or canaloplasty combined with phacoemulsification. This retrospective study evaluates the results between two groups in three IOP ranges, drug therapy reduction, intraoperative complications and additional IOP lowering methods including surgery in a 12-month follow-up.

## Methods

Between 2010 and 2013, a total of 439 patients already diagnosed with POAG were referred to our clinic for surgery due to uncontrolled IOP despite maximal local and systemic medication, progression of visual field defects or drug therapy intolerance. A total of 602 eyes were initially evaluated and were scheduled to undergo canaloplasty (Group A) or canaloplasty combined with phaco (Group B) due to lens opacity. All patients underwent a baseline standard ophthalmic examination including ophthalmic history with previous ocular surgeries or laser treatments, glaucoma medication use, Humphrey visual field 30–2 SITA Fast test, IOP measurement using Goldmann applanation tonometry, visual acuity testing in decimal Snellen, slit-lamp biomicroscopy for anterior segment evaluation, gonioscopy with angle grading and indirect ophthalmoscopy for fundus assessment. The two main groups were then divided according to the Hodapp, Parrish, Anderson (H-P-A) classification system [9], into three subgroups. Demographics are presented in Table 1. All patients were operated on St. Johannes Hospital Eye Clinic, Dortmund, Germany by MK and written consent was obtained prior to surgery. The tenets of the Declaration of Helsinki were fully respected. The operation was performed ab externo by placing the first flap at 11 o’clock regardless of left or right eye. After preparation of the second scleral flap, access to Schlemm’s canal was gained and a microcatheter iTRACK™ 250 (Ellex Medical Lasers Ltd., 3–4 Second Avenue, Mawson Lakes, SA 5095 Australia) was inserted. Dilation was performed through the injection of sodium hyaluronate, Healon GV®, 0.85 ml from Abbott (Abbott laboratories, Lake Bluff, Illinois, United States). The microcatheter was then removed and tensioning of the canal with a 10.0 polypropylene suture was performed. Eighteen eyes from both groups were converted to other glaucoma surgery intraoperative due to incomplete cannulation and were excluded from the analysis (Table 1.) Patients remained in our clinic and were discharged at 1.51 ± 0.5 days for Group A and 1.48 ± 0.5 days for Group B. The post-operative regimen was antibiotics combined with corticosteroids eye drop solution 5 times daily and eye ointment 1 time daily, replaced after 2 weeks with non-corticosteroid anti-inflammatory agents 5 times daily for 2 weeks. Follow up took place at 1 day, 2 weeks, 1 month, 3 months, 6 months and 12 months. Postoperative follow up checks included IOP measurement using Goldmann applanation tonometry, visual acuity testing in decimal Snellen, slit-lamp biomicroscopy for anterior segment evaluation and assessment of complications with decision of subsequent surgical interventions. Humphrey visual field 30–2 SITA Fast test was repeated again in 12 months. When additional surgery was required, full thickness opening surgery or transscleral diode cyclophotocoagulation were performed due to inadequate IOP lowering effect, failure to comply with medical therapy or both. We did not perform YAG - Goniopuncture in any of the 584 eyes. Trabeculectomy scleral flap was created at 2 o’clock and was combined with mitomycin 0.02% and ologen® Collagen Matrix (Aeon Astron USA Inc.) implantation. Ologen is a biodegradable and implantable porcine extracellular matrix made of atelocollagen cross-linked with glycosaminoglycan, specifically configured to support and modulate tissue repair processes in connective and epithelial ocular tissues. Transcleral diode cyclophotocoagulation was performed in danger of wipe out syndrome or when conjunctiva was limiting the success of full thickness opening surgery. Complete success was regarded as obtaining postoperative IOP ≤ 21 mmHg, ≤ 18 mmHg, and ≤ 16 mmHg without medical therapy and partial success an IOP ≤ 21 mmHg, ≤ 18 mmHg, and ≤ 16 mmHg with medical therapy. Success is reported in the percentage of eyes reaching these goals using the Kaplan Meier test. IOP percentage reduction and medical therapy percentage reduction to baseline was also evaluated. MedCalc was used for statistical purposes and significance was given at *p* < 0.05. Regarding distribution, Kolmogorov-Smirnov and chi-squared test were performed. Follow up duration was 12 months. The local institutional review board committee approved this retrospective study.

**Table 1:** Demographics divided into group A and B and subgroups

Demographics	n
No. of patients	439
No. of females patients	246
Age (years)	72.89 ± 11.99
No. of male patients	193
Age (years)	72.07 ± 12.12
Bilateral treatment	167
Unilateral treatment	268
No. of eyes operated total	602
No. of eyes excluded due to intraoperative conversion to other surgery	18
→ Trabeculectomy with ologen and MMC 0.02%	9
→ Trabeculectomy with ologen and MMC 0.02% and Phaco	7
→ Viscocanalostomy	2
No. of studied eyes	584
Group A	n
Canaloplasty	262
→ Subgroup (Advanced POAG – target IOP ≤ 16 mmHg)	172
• MD – dB	14.31 ± 3.56
• IOP – mmHg	19.15 ± 6.42
• Antiglaucoma agents	2.71 ± 0.95
• DCVA – Snellen	0.58 ± 0.29
→ Subgroup (Moderate POAG – target IOP ≤ 18 mmHg)	51
• MD – dB	7.31 ± 3.22
• IOP – mmHg	20.66 ± 5.04
• Antiglaucoma agents	2.77 ± 0.94
• DCVA – Snellen	0.79 ± 0.21
→ Subgroup (Early POAG – target IOP ≤ 21 mmHg)	39
• MD – dB	4.22 ± 4.13
• IOP – mmHg	21.25 ± 5.67
• Antiglaucoma agents	2.69 ± 1.21
• DCVA – Snellen	0.64 ± 0.21
Group B	n
Canaloplasty with Phacoemulsification	322
→ Subgroup (Advanced POAG – target IOP ≤ 16 mmHg)	212
• MD – dB	16.11 ± 4.42
• IOP – mmHg	19.39 ± 7.49
• Antiglaucoma agents	2.70 ± 0.95
• DCVA – Snellen	0.53 ± 0.25
→ Subgroup (Moderate POAG – target IOP ≤ 18 mmHg)	51
• MD – dB	8.01 ± 3.01
• IOP – mmHg	19.54 ± 5.90
• Antiglaucoma agents	2.32 ± 1.02
• DCVA – Snellen	0.56 ± 0.26
→ Subgroup (Early POAG – target IOP ≤ 21 mmHg)	39
• MD – dB	5.06 ± 2.17
• IOP – mmHg	19.42 ± 7.32
• Antiglaucoma agents	2.40 ± 1.06
• DCVA – Snellen	0.55 ± 0.23

*POAG* = primary open angle glaucoma; *IOP* = intraocular pressure; *MD* = mean deviation; *dB* = decibels; *DCVA* = distance corrected visual acuity

## Results

**Fig. 1:**
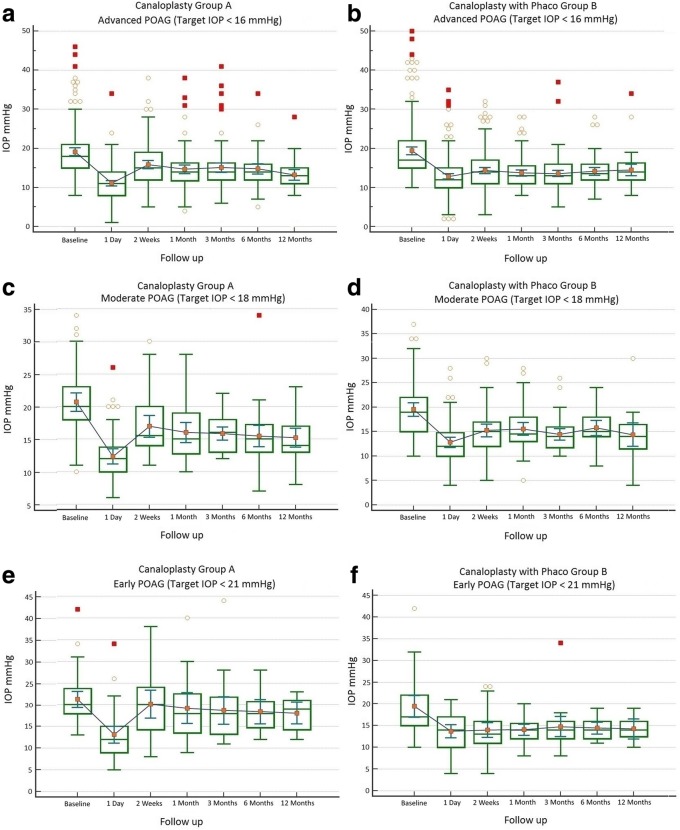
Reduction of mean IOP from baseline throughout the follow up period. **a** Group A IOP ≤ 16 mmHg; **b** Group B IOP ≤ 16 mmHg; **c** Group A IOP ≤ 18 mmHg; **d** Group B IOP ≤ 18 mmHg; **e** Group A IOP ≤ 21 mmHg; **f** Group B IOP ≤ 21 mmHg

**Fig. 2:**
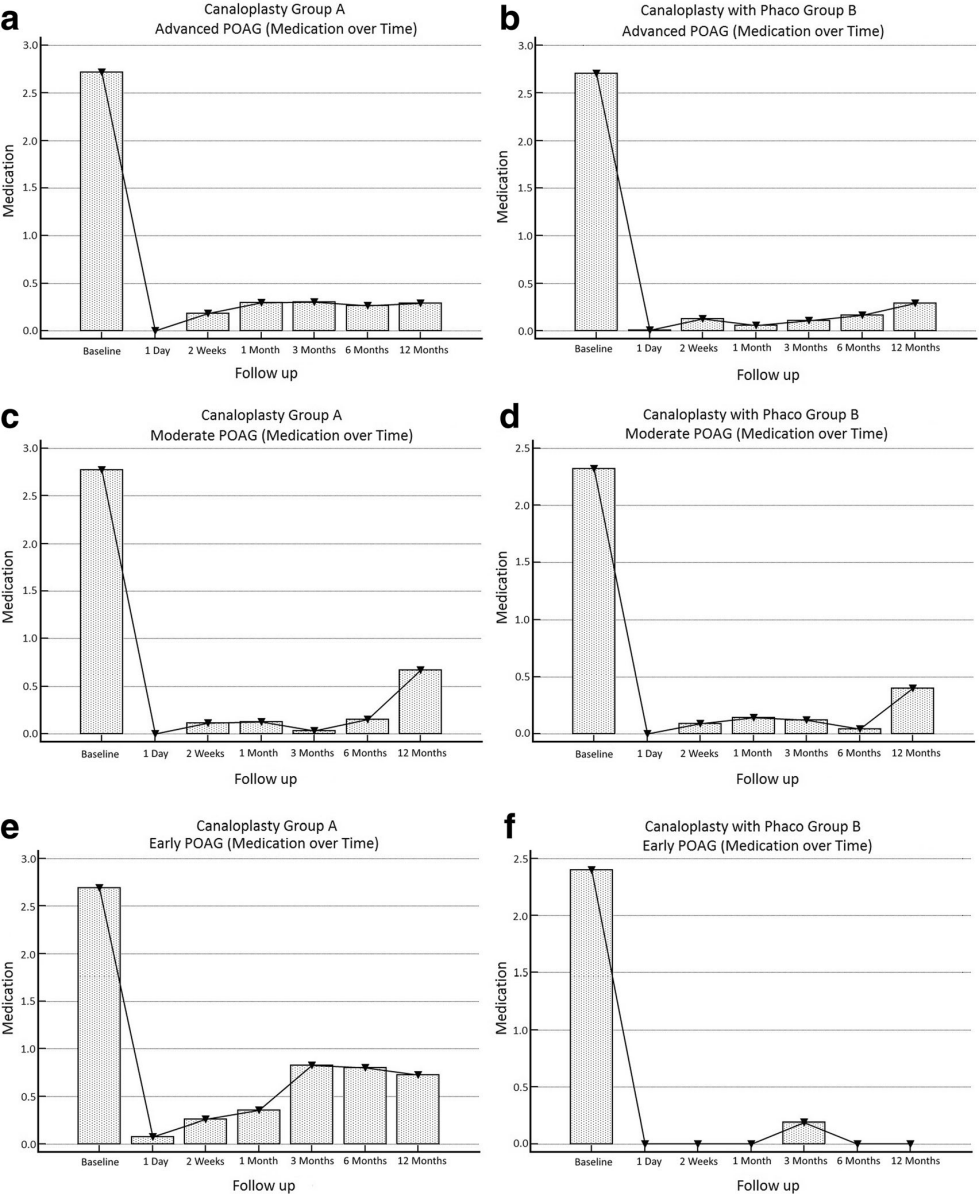
Reduction of mean antiglaucoma agents from baseline throughout the follow up period. **a** Group A (advanced POAG - IOP ≤ 16 mmHg); **b** Group B (advanced POAG - IOP ≤ 16 mmHg); **c** Group A (moderate POAG - IOP ≤ 18 mmHg); **d** Group B (moderate POAG - IOP ≤ 18 mmHg); **e** Group A (early POAG - IOP ≤ 21 mmHg); **f** Group B (early POAG - IOP ≤ 21 mmHg)

**Fig. 3:**
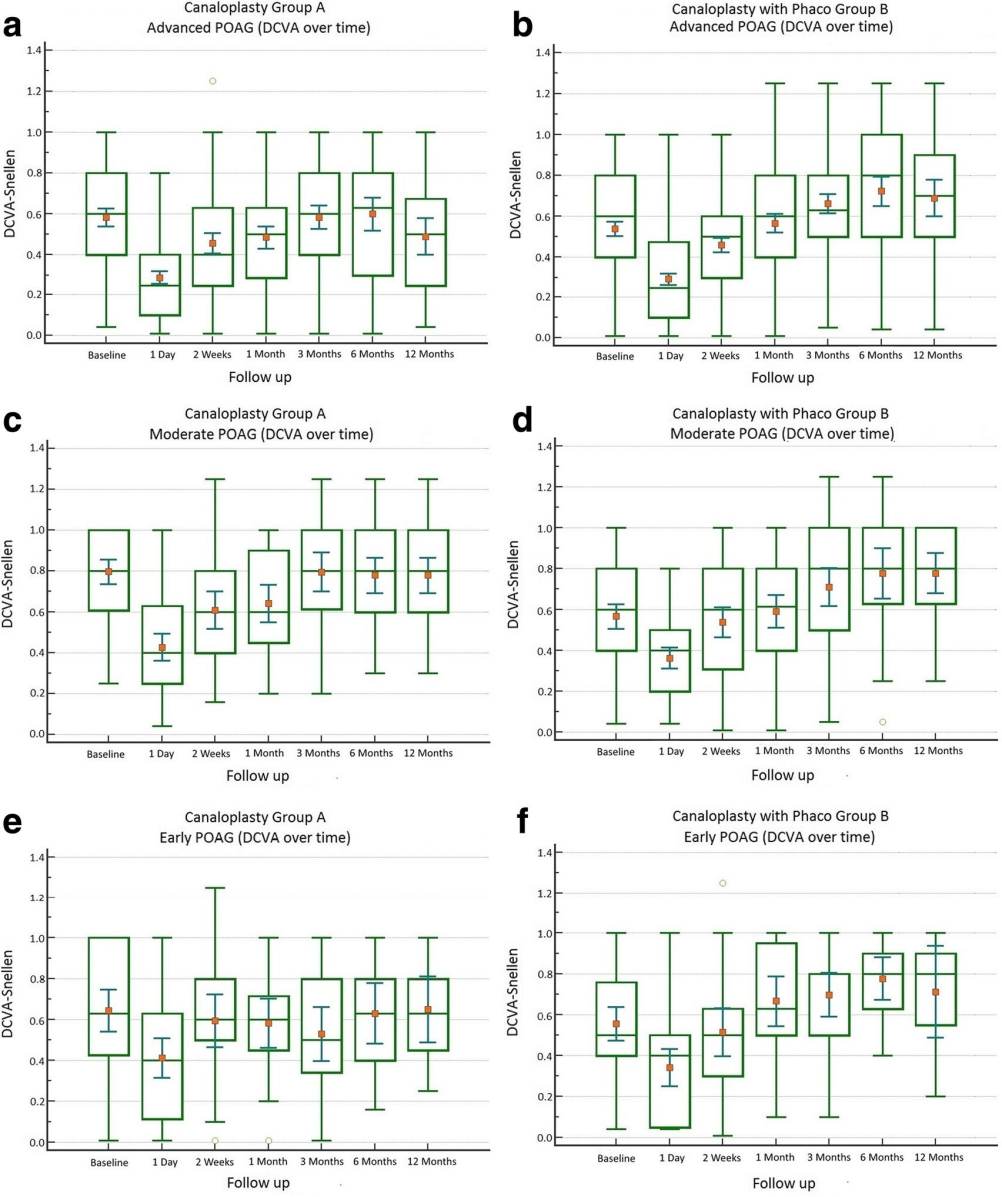
Fluctuation of mean DCVA in Snellen from baseline throughout the follow up period. **a** Group A (advanced POAG - IOP ≤ 16 mmHg); **b** Group B (advanced POAG - IOP ≤ 16 mmHg); **c** Group A (moderate POAG - IOP ≤ 18 mmHg); **d** Group B (moderate POAG - IOP ≤ 18 mmHg); **e** Group A (early POAG - IOP ≤ 21 mmHg); **f** Group B (early POAG - IOP ≤ 21 mmHg)

**Fig. 4:**
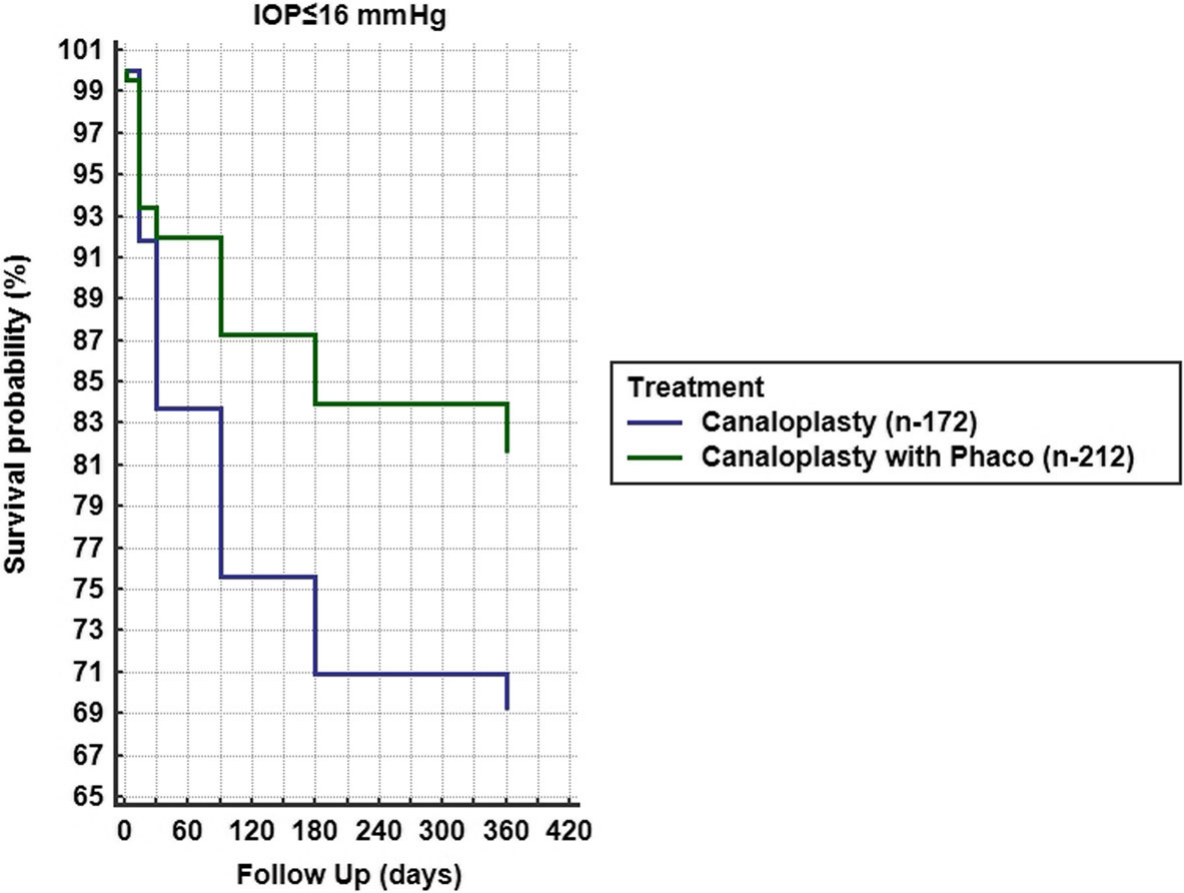
Kaplan Meier between Groups A and B of complete success, (IOP ≤ 16 mmHg, Log rank test *p* = 0.0041)

**Fig. 5:**
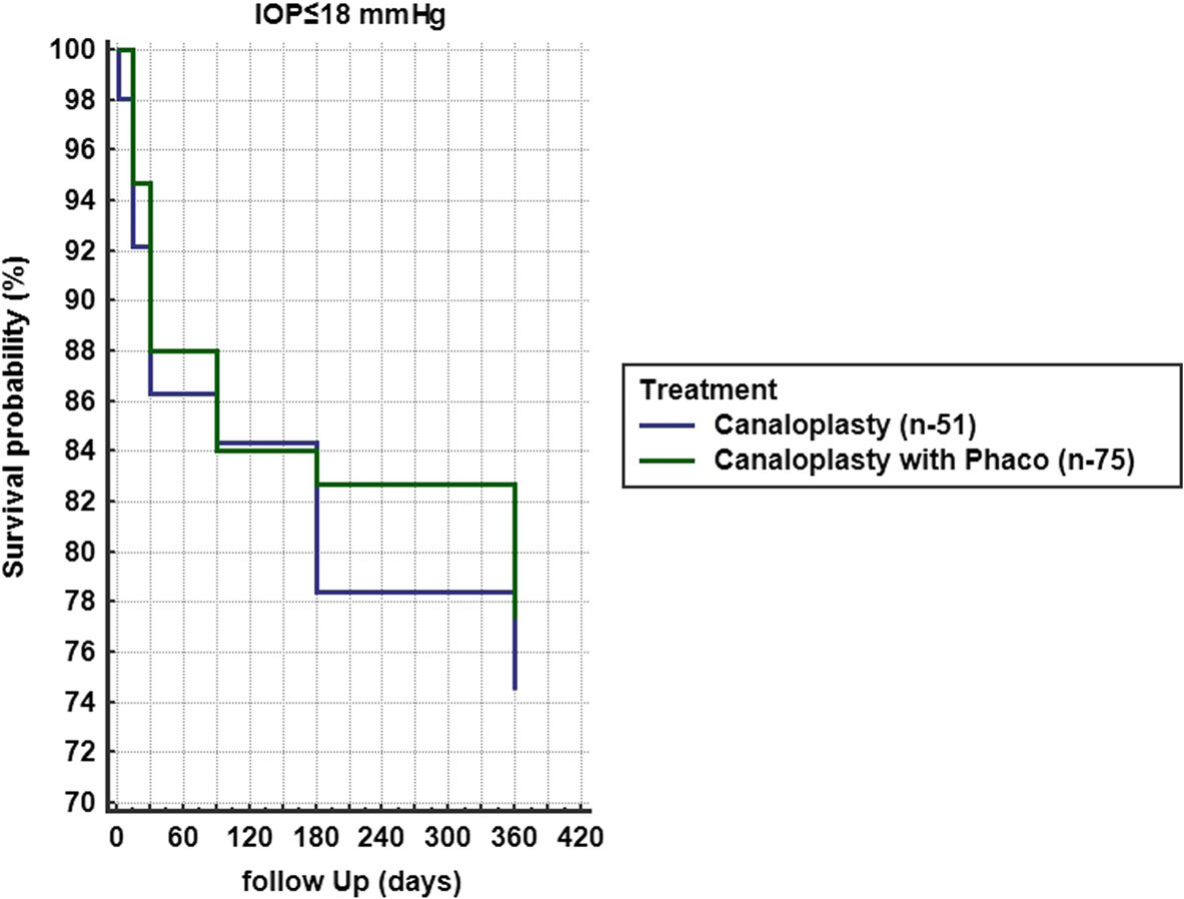
Kaplan Meier between Groups A and B of complete success, (IOP ≤ 18 mmHg, Log rank test *p* = 0.6935)

A total of 18 eyes (2.99%) were converted intraoperatively to other procedures and were excluded from the analysis. In total, 584 eyes were analyzed (Table 1). Hyphema, descemet membrane detachment, transient IOP raise or hypotony were among the post-operative complications (Table 2). Mean IOP baseline for group A with target IOP ≤ 16 mmHg was 19.15 ± 6.42 mmHg with 2.71 ± 0.95 antiglaucoma agents and reduced at 12 months to 13.26 ± 4.50 mmHg with 0.28 ± 0.99 agents (Figs. 1 a and 2 a). Medication reduction reached 91.43 ± 30.74% (Table 3) and IOP reduction reached 27.88 ± 29.50% at 12 months. By the end of the follow up, complete success was achieved in 69.19% and partial success in 95.23% of cases. Additional surgery was performed in 9 eyes (Table 2). Mean IOP of Group B with target IOP ≤ 16 mmHg was baseline 19.39 ± 7.49 mmHg with 2.70 ± 0.95 antiglaucoma agents and reduced at 12 months to 14.51 ± 4.69 mmHg with 0.29 ± 0.87 (Figs. 1 b and 2 b). Medication reduction reached 89.43 ± 29.28% (Table 3) and IOP reduction reached 16.52 ± 25.37% at 12 months. By the end of follow up, complete success was achieved in 81.60% and partial success in 92.26%. Additional surgery was performed in 10 eyes (Table 2). Mean IOP and medication reduction over the 12-month follow-up for both groups with target IOP subgroups was adequate (Figs. 1 a-f and 2 a-f, Table 3). Mean distance corrected visual acuity (DCVA) in group A was stable except from the first day to the first month in most subgroups. Some fluctuations in DCVA between the follow up were due to ocular surface problems (Fig. 3 a,c,e). In group B and subgroups DCVA compared to baseline increased significantly (Fig. 3 b,d,f). Kaplan Meier Log rank test was statistically significant between Group A and B for target IOP ≤ 16 mmHg and target IOP ≤ 21 mmHg (*p* = 0.0041 and *p* = 0.0312 respectively) (Fig. 4) but was not significant for target IOP ≤ 18 mmHg (*p* = 0.6935), (Fig. 5). Complete success for IOP ≤ 18 mmHg was achieved in 74.51% in Group A and in 77.33% in Group B. Respectively, partial success was 91.66 and 90.47%. In target IOP ≤ 21 mmHg, complete success for Groups A and B were 74.31 and 83.33% and partial success 90.00 and 93.10%, respectively. Humphrey visual field 30–2 SITA Fast test at 12 months did not show progression in main groups and subgroups and was not statistically significant (Wilcoxon test *p* = 0.2541, *p* < 0.3256 and *p* = 0.4532, respectively).

**Table 2:** Complications divided into group A and B and subgroups

Post operative complications		
Group A	n	%
Canaloplasty	262	
Microhyphema (< 1.0 mm)	25	9.54
Macrohyphema (> 1.0 mm)	3	1.14
Descemet membrane detachment (DMD)	6	2.29
Hemorrhagic DMD	0	0
Cheese wiring	1	0.38
Anterior chamber inflammation	0	0
Subgroup (Advanced POAG – target IOP ≤ 16 mmHg)	172	
Transient hypotony (< 5 mmHg)	3	1.74
Transient hypertony (> 20 mmHg)	11	6.39
Additional surgery (Trabeculectomy with MMC 0.02% and ologen)	9	5.23
Additional surgery (Cyclophotociagulation)	2	1.16
Subgroup (Moderate POAG – target IOP ≤ 18 mmHg)	51	
Transient hypotony (< 5 mmHg)	0	0
Transient hypertony (> 20 mmHg)	2	3.91
Additional surgery (Trabeculectomy with MMC 0.02% and ologen)	1	1.96
Additional surgery (Cyclophotociagulation)	0	0
Subgroup (Early POAG – target IOP ≤ 21 mmHg)	39	
Transient hypotony (< 5 mmHg)	0	0
Transient hypertony (> 25 mmHg)	6	15.38
Additional surgery (Trabeculectomy with MMC 0.02% and ologen)	0	0
Additional surgery (Cyclophotociagulation)	0	0
Group B	n	%
Canaloplasty with Phacoemulsification	322	
Microhyphema (< 1.0 mm)	13	4.03
Macrohyphema (> 1.0 mm)	2	0.62
Descemet membrane detachment (DMD)	6	1.86
Hemorrhagic DMD	1	0.31
Cheese wiring	0	0
Anterior chamber inflammation	8	2.48
Subgroup (Advanced POAG – target IOP ≤ 16 mmHg)	212	
Transient hypotony (< 5 mmHg)	3	1.41
Transient hypertony (> 20 mmHg)	22	10.37
Additional surgery (Trabeculectomy with MMC 0.02% and ologen)	8	3.77
Additional surgery (Cyclophotociagulation)	2	0.62
Subgroup (Moderate POAG – target IOP ≤ 18 mmHg)	75	
Transient hypotony (< 5 mmHg)	1	1.33
Transient hypertony (> 20 mmHg)	8	10.66
Additional surgery (Trabeculectomy with MMC 0.02% and ologen)	1	1.33
Additional surgery (Cyclophotociagulation)	0	0
Subgroup (Early POAG – target IOP ≤ 21 mmHg)	35	
Transient hypotony (< 5 mmHg)	1	2.85
Transient hypertony (> 25 mmHg)	3	8.57
Additional surgery (Trabeculectomy with MMC 0.02% and ologen)	0	0
Additional surgery (Cyclophotociagulation)	0	0

*POAG* = primary open angle glaucoma; *IOP* = intraocular pressure

**Table 3:** Paired samples *t*-test of medication percentage reduction in comparison to baseline divided into groups A and B and subgroups throughout the follow up period

Group A – Medication %↓ Advanced POAG	n	Mean	Range	SD	Different (*P* < 0.05) from factor nr
(1) A) Baseline	172	0.00	0.0	±0.00	(2)(3)(4)(5)(6)(7)
(2) B) 1 Day	172	− 100.00	−100.0 / -100.0	±0.00	(1)(3)(4)(5)(6)(7)
(3) C) 2 Weeks	171	−93.59	−100.0 / + 50.0	±19.94	(1)(2)
(4) D) 1 Month	161	−90.38	−100.0 / + 50.0	±26.26	(1)(2)
(5) E) 3 Months	155	−89.60	−100.0 / + 50.0	±29.68	(1)(2)
(6) F) 6 Months	151	−89.40	−100.0 / -100.0	±33.84	(1)(2)
(7) G) 12 Months	168	−91.43	−100.0 / + 66.0	±30.74	(1)(2)
Group B - Medication %↓ Advanced POAG	n	Mean	Range	SD	Different (P < 0.05) from factor nr
(1) A) Baseline	212	0.00	0,0	±0.00	(2)(3)(4)(5)(6)(7)
(2) B) 1 Day	212	−99.90	−100.0 / -80.0	±1.37	(1)(3)(5)(6)(7)
(3) C) 2 Weeks	187	−95.56	−100.0 / + 33.0	±18.37	(1)(2)(7)
(4) D) 1 Month	173	−97.56	−100.0 / 0.0	±15.48	(1)(7)
(5) E) 3 Months	134	−95.83	−100.0 / 0.0	±15.85	(1)(2)
(6) F) 6 Months	132	−93.78	−100.0 / 0.0	±21.28	(1)(2)
(7) G) 12 Months	168	−89.43	−100.0 / 00	±29.28	(1)(2)(3)(4)
Group A - Medication %↓ Moderate POAG	n	Mean	Range	SD	Different (P < 0.05) from factor nr
(1) A) Baseline	51	0.00	0.0	±0.00	(2)(3)(4)(5)(6)(7)
(2) B) 1 Day	51	−100.00	−100.0 / -100.0	±0.00	(1)(7)
(3) C) 2 Weeks	50	−95.45	−100.0 / -33.0	±15.17	(1)(7)
(4) D) 1 Month	48	−95.28	− 100.0 / -33.0	±15.46	(1)(7)
(5) E) 3 Months	41	−96.55	−100.0 / 0.0	±18.56	(1)(7)
(6) F) 6 Months	39	−95.96	−100.0 / -66.0	±10.73	(1)(7)
(7) G) 12 Months	48	−76.66	−100.0 / + 100.0	±55.51	(1)(2)(3)(4)(5)(6)
Group B - Medication %↓ Moderate POAG	n	Mean	Range	SD	Different (P < 0.05) from factor nr
(1) A) Baseline	75	0.00	0.0	±0.00	(2)(3)(4)(5)(6)(7)
(2) B) 1 Day	75	−100.00	−100.0 / -100.0	±0.00	(1)(7)
(3) C) 2 Weeks	68	−96.54	−100.0 / -50.0	±12.38	(1)(7)
(4) D) 1 Month	60	−94.14	−100.0 / 0.0	±19.42	(1)(7)
(5) E) 3 Months	55	−94.30	−100.0 / 0.0	±22.23	(1)(7)
(6) F) 6 Months	49	−98.48	−100.0 / -66.0	±7.10	(1)(7)
(7) G) 12 Months	63	−84.21	−100.0 / 0.0	±34.00	(1)(2)(3)(4)(5)(6)
Group A - Medication %↓ Early POAG	n	Mean	Range	SD	Different (P < 0.05) from factor nr
(1) A) Baseline	39	0.00	0.0	±0.00	(2)(3)(4)(5)(6)(7)
(2) B) 1 Day	39	−96.15	−100.0 / + 50.0	±24.01	(1)(5)(7)
(3) C) 2 Weeks	39	−92.85	−100.0 / -50.0	±15.43	(1)
(4) D) 1 Month	34	−86.76	−100.0 / 0.0	±28.72	(1)
(5) E) 3 Months	35	−72.77	−100.0 / + 100.0	±50.58	(1)(2)
(6) F) 6 Months	26	−77.17	−100.0 / -20.0	±33.04	(1)
(7) G) 12 Months	30	−65.15	−100.0 / + 50.0	±50.80	(1)(2)
Group B - Medication %↓ Early POAG	n	Mean	Range	SD	Different (P < 0.05) from factor nr
(1) A) Baseline	35	0.00	0,0	±0.00	(2)(3)(4)(5)(6)(7)
(2) B) 1 Day	35	−100.00	−100.0 / -100.0	±0.00	(1)(5)
(3) C) 2 Weeks	30	−100.00	−100.0 / -100.0	±0.00	(1)(5)
(4) D) 1 Month	24	−100.00	−100.0 / -100.0	±0.00	(1)(5)
(5) E) 3 Months	26	−95.00	−100.0 / -50.0	±15.38	(1)(2)(3)(4)(6)(7)
(6) F) 6 Months	28	−100.00	−100.0 / -100.0	±0.00	(1)(5)
(7) G) 12 Months	29	−100.00	−100.0 / -100.0	±0.00	(1)(5)

*POAG* = primary open angle glaucoma; *SD* = standard deviation; *nr* = number

↓Reduction of medication percentage

## Discussion

With the advent of canaloplasty in the last decade as a new surgical option in managing IOP in glaucoma patients, an increasing number of surgeons have started to favor this approach for its safety, efficacy and overall good results. Canaloplasty lowers IOP by a 360-degree viscodilation and tensioning of the Schlemm canal. This procedure is still less likely to be successful in eyes with a nonreversible collapse of collector channels or other outflow pathways that cannot be mechanically enlarged [10]. Episcleral venous pressure and transtrabecular diffusion plays a significant role in the outcomes of canaloplasty procedure. Chanelography and provocative gonioscopy can be used as a tool to predict the outcomes preoperatively [11]. Overall, the efficacy and safety of canaloplasty is well documented [12–19]. In 2011, Grieshaber et al. published the results of a prospective study of 32 White patients with POAG in which the mean IOP dropped from 27.3 ± 5.6 mmHg preoperatively to 12.8 ± 1.5 mmHg at 12 months and 13.1 ± 1.2 mmHg at 18 months [12]. They reported a complete success rate for IOP ≤ 21 mmHg, ≤ 18 mmHg and ≤ 16 mmHg of 93.8, 84.4 and 74.9%, respectively at 12 months. The same group studied in the same manner African patients and reported a mean IOP at 12 months of 15.4 ± 5.2 mmHg from baseline of 45.0 ± 12.1 mmHg, reporting a complete success rate for IOP ≤ 21 mmHg of 77.5% and qualified success rate of 81.6% at 36 months [13]. In our study, group A showed similar results regarding the mean IOP, which reduced from baseline 19.15 ± 6.42 to 13.26 ± 4.50 mmHg for IOP ≤ 16 mmHg, 20.66 ± 5.04 to 15.19 ± 3.97 mmHg for IOP ≤ 18 mmHg and 21.25 ± 5.67 to 18.09 ± 3.75 mmHg for IOP ≤ 21 mmHg. However, in our study, standard deviation (SD) of mean IOP was significantly higher and our complete success rate was significantly lower with 69.19% for IOP ≤ 16 mmHg, 74.51% for IOP ≤ 18 mmHg and 74.31% for IOP ≤ 21 mmHg. Our partial success rate was significantly higher with 95.23, 91.66 and 90.00%, respectively, and we had a significant reduction of the need for medication in all three subgroups. Bull et al. reported 3-year results investigating the safety and efficacy of canaloplasty in a prospective, multi-center, interventional study of 109 eyes of 109 adult, open-angle glaucoma patients undergoing canaloplasty or combined cataract-canaloplasty surgery. Qualifying preoperative IOPs were at least 16 mmHg with historical IOPs of at least 21 mmHg with or without medical therapy. Eyes with canaloplasty showed a mean baseline IOP of 23.0 ± 4.3 mmHg and mean glaucoma medication usage of 1.9 ± 0.7 medications, which decreased to a mean IOP of 15.1 ± 3.1 mmHg on 0.9 ± 0.9 medications at 3 years postoperatively. Eyes with combined cataract-canaloplasty surgery showed a mean baseline IOP of 24.3 ± 6.0 mmHg on 1.5 ± 1.2 medications, which decreased to a mean IOP of 13.8 ± 3.2 mmHg on 0.5 ± 0.7 medications at 3 years. Intraocular pressure and medication use results for all study eyes were significantly decreased from baseline (*p* < 0.00001) at all intervals. Late postoperative complications included cataracts (19.1%) and transient IOP elevation (1.8%) [14]. We demonstrate similar results in groups A and B in terms of mean IOP reduction and mean medication reduction (Table 3). We also reported transient IOP elevation in 19 eyes group A (7.25%) and group B in 33 eyes (10.24%). These eyes where either treated with medication or with additional surgery if IOP was not manageable (Table 2). Cataract formation following canaloplasty was not reported in group A. Brusini et al. [15, 16] reported the midterm results of a multicenter prospective study of 218 eyes from 197 POAG patients with a 23.1 ± 10.6 month follow up. The mean IOP reduction reported was 44% from baseline. After 2 years of follow-up, a partial success rate based on postoperative IOP ≤ 21 mmHg, ≤ 18 mmHg, and ≤ 16 mmHg was 92.1, 84.3, and 68.5%, respectively, and a complete success for an IOP ≤ 21 mmHg, ≤ 18 mmHg, and ≤ 16 mmHg 70.8, 67.4, and 59.5%, respectively. The number of medications used preoperatively and at the 2-year follow-up was 3.2 ± 0.9 and 1.1 ± 1.3, respectively. They reported incomplete operations in 20 eyes (9.2%), hyphema in 47 eyes (23.7%), Descemet membrane detachment in 11 eyes (5.5%), and IOP spikes > 10 mmHg in 12 cases (6.1%). We reported similar results in both groups A and B for IOP ≤ 21 mmHg, ≤ 18 mmHg, and ≤ 16 mmHg, however our follow up was significantly shorter. We excluded 18 eyes originally from the analysis due to incomplete cannulation of Schlemm’s canal and conversion to other glaucoma surgery. We had similar post-operative complications but reported over a shorter follow up period. To our knowledge, this study, despite its retrospective nature and the lack of a control group, reports on the largest cohort of eyes that underwent canaloplasty or canaloplasty combined with phacoemulsification. Furthermore, it presents an interesting mix of results that possibly is more reflective of real world practice in comparison to similar studies despite our smaller follow up. Our partial success rate was above 90% in both groups and overall we demonstrated an adequate reduction of mean IOP and reduced post-operative need for medication. Our percentage reduction of IOP was high at 1 day post-op and gradually reduced with one group showing 8% reduction while other groups showing 16 to 17% and some groups even above 25%. This could be explained from the low baseline IOP in all our groups that were receiving at baseline up to 5 antiglaucoma agents to maintain IOP, in particular for group A (IOP ≤ 21 mmHg) whose baseline mean IOP with medication was 21.25 ± 5.67 mmHg. The results of this group in comparison to all other groups in all parameters were inferior. We believe that this interesting result was due to failure of collector channels enlargement possibly resulting from increased episcleral venous pressure and decreased transtrabecular diffusion. Nonetheless, the mean IOP of this group was below its target and the medication reduced from 2.69 ± 1.2 to 0.72 ± 1.0 at 12 months, a reduction of 65.15 ± 50.80% from baseline. Between groups A and B, the results were superior in most parameters for group B, showing that canaloplasty with phaco provides better outcomes as shown in other studies [14, 17–19]. Overall, we reported acceptable complete and partial success rates and managed to surgically reduce IOP with minor complications and a few additional surgical interventions.

## Conclusions

Overall, canaloplasty alone and canaloplasty combined with phacoemulsification are safe surgical procedures that significantly reduce IOP in POAG. An incomplete intraoperative cannulation of Schlemm’s canal can result in conversion to other glaucoma surgery. The most common post-operative complications are hyphema, DMD, hypotony and transient IOP elevation that requires additional medication and possibly surgical intervention if medication fails. Canaloplasty combined with phacoemulsification provided superior results in terms of achieving the target IOP with no additional medication in comparison to canaloplasty alone.
